# An optimal investment consumption model for retirees with no health insurance

**DOI:** 10.1016/j.heliyon.2024.e29034

**Published:** 2024-04-09

**Authors:** Nelson Dzupire, John Mutepuwa

**Affiliations:** a School of Natural and Applied Sciences, University of Malawi, P.O. Box 280, Zomba, Malawi; b School of Science and Technology, Department of Mathematical Sciences, Malawi University of Business and Applied Sciences, Private Bag 303, Chichiri, Blantyre 3, Malawi

**Keywords:** Stochastic differential equations, Hamilton Jacobi Bellman equation, Utility function, Partial differential equation, Bellman's optimality principle, Health expenditure, Sensitivity analysis

## Abstract

Retirees meet a number of problems as they are growing older which needs persistent attention. Hence, without a doubt, the outcomes of the financial markets influence the choices that people make when nearing retirement. In our model, the stock price dynamics follow Geometric Brownian motion (GBM) and our goal was to optimize the expected discounted utility of consumption and terminal wealth whilst considering health expenses. The investment return process comprises risk free asset and risky assets, and the health expenses. We choose power utility functions where comprehensive solutions for Hyperbolic Absolute Risk Aversion (HARA) utility functions are obtained and optimal investment, consumption and health expenditure strategies are derived by applying dynamic programming and variable change technique on the Hamilton-Jacobi-Bellman (HJB) equations. In our numerical results it showed various effects of some economic and market parameters on the optimal investment, consumption and health expense strategies. The inflation price market risk governs the amount invested in stock, bond and also how much to be put in health to sustain a given period of the retiree's lifetime. As the health welfare rate R increases, the proportion of wealth invested in the stock increases. We also investigated the effects of the high correlation coefficients and low correlation coefficients on consumption and income rate respectively. As the constant variance discounting coefficient increases, seasoned enterprise annuity retirees decrease their allocation to the risky assets. Finally, a numerical example is presented to depict the effects of financial parameters on the optimal investment strategy with health expenditure.

## 1 Introduction

Retirement has been an issue needing attention and it has been an outstanding issue of discussion for the past decades in low-income countries. Nevertheless, there is no model for retirees that face health conditions with no health insurance. Numerous research papers extensively explore optimal investment and consumption models, emphasizing the use of a house as collateral to determine borrowing amounts [8]. Related studies for retirees to make decisions on how to allocate assets on the financial market have considered housing as a major factor which determine consumption leaving out health expenditure [8], [18], [19]. Retirement is the act of leaving employment at a designated age or for different reasons [2]. Retirement usually refers to the situation of not being paid at work. According to Atchley [2], retirement is one of the main modifications in life that make the individual to leave one part of the life and join the other way of life. This transformation often affects many life domains and is often accompanied by a decline in life satisfaction, self-evaluation and quality of life [12]. Different people think of retirement in many and different ways. Bur [6] viewed retirement as the circumstance in which an individual departs from their employment either by doing it willingly or under obligation, following the completion of a designated duration of service or due to being compelled to retire by reasons such as compulsory retirement, lay-off, dismissal (resulting from acts of insubordination or misconduct), illness, incapacity, or personal choice to withdraw from the position. Nwachukwu [13], on his part, considered retirement as a socially accepted means of withdrawing from one's occupation or business in later life to enjoy leisure, freedom or simply to survive with health problems. As Jonathan Clements, a personal finance columnist with the Wall Street Journal succinctly puts it, “Retirement is like a long vacation.” The goal is to enjoy it to the fullest, but not so fully that you run out of money [13].

Retirement can be in many forms. Bur [6] recognized diverse origins of retirement such as voluntary and involuntary retirement, lay-offs, flexible retirement, redundancy, discharge of staff, termination of appointment and dismissal. Oyuke [14] highlighted that regardless of the core and importance of retirement, preparation towards retirement by most workers is not enough yet. The main aim of retirement preparation programs is to enable a worker from attaining a practical understanding of life after retirement to alleviate concerns surrounding this phase. To say it in other ways, it aims at enhancing prospective retirees' adaptation to retirement and to provide assistance in managing the new phase of life [12]. However, with several younger generations approaching retirement, prioritizing the development of products that guarantee lifelong financial security for retirees has become a forefront concern within the financial industry. In public policy, there is active debate on whether the social security system can be changed to improve the welfare of present and future retirees. Despite all these interests, very little is understood about the asset allocation in terms of decisions for retirees [20]. Retirement is connected with significant individual choices like spending habits, healthcare costs, and investments, as well as broader policy considerations which encompass aspects such as insurance, pensions, and the distinction between compulsory and voluntary retirement [4]. The big stock market boom between 1995 and 2000 led to an imaginable increase in the number of people who chose voluntary early retirement [9]. In reverse antecedent, these people's choices on retirement, consumption and savings are expected to have a significant impact on aggregate consumption and investments, and to have a large effect on the world's financial markets and economy [5].

Pension systems pose particular sensitivity, especially in low-income countries, where a majority of workers lack substantial retirement benefits and struggle to accumulate enough earnings throughout their careers to support their retirement phase [1]. The lifestyle of many employees depends to a large extent on many factors, some of which include an individual's culture, inclinations, available resources, and the economic and socio-environmental conditions in their surroundings. The issue of retirement has been very interesting where attention has been increased in many organizations in developing countries. According to Nwachukwu [13] many factors account for this refreshed emphasis, which include: Firstly, any employee is expected to work throughout his/her entire life on earth; arrangements must be made for declining years. Secondly, the life expectancy of many workers has been increased and many of the retirees are anticipated to work until they retire. Even those who believe that death could come at any time harbor the ambition that their retirement benefits will be given to their next of kin. The extended family system, which in the past assisted retirees, is gradually losing its impact to Western culture and influence. There is increasing awareness and emphasis that people, regardless of age, should learn to be self-supporting. Finally, the government has enacted laws motivating employers to pay retirement benefits and gratuities to certified retirees.

While numerous state and local government employers permit retirees to maintain their enrollment in employee health plans, the pervasiveness of retiree health insurance (RHI) is waning in various sectors where this benefit was once widespread. According to Franzel & Brown [10], there are meaningful factors that impact firms to eliminate the RHI benefit. These factors include the continuing rapid increase in the cost of health insurance combined with the old age of the individuals. Additionally, the increasing durability of retirees results in more retirees relative to active workers and increases the total cost.

Nonetheless, there is very little work that has been examined on allocating assets in retirement when households face health risk. Cocco [8] Constructed a model outlining the ideal portfolio and consumption choices for a standard investor, facing uncertain labor income over time. In his model, Cocco [8] found out that the investor needs to decide the size of the house to buy, the amount of money needed to consume goods, the amount of money to borrow using the house as a collateral and portfolio composition among stocks and treasury bills. Furthermore, Yao and Zhang [19] examined the model for the optimal dynamic portfolio decisions for investors who acquire housing services from renting or owning a house. Aydilek [3] constructed an optimization model of retiree decisions under recursive utility with housing. Therefore, we develop and theoretically examine an optimal mathematical model for investment and consumption for retirees without health insurance

The rest of the paper is organized as follows. The proposed model is formulated and analyzed in Section 2. In Section 3, qualitative analysis is performed while in Section 4, sensitivity analysis is performed to illustrate the effect of market parameters on the optimal investment consumption strategies under HARA utility (Power utility case). Numerical simulations are also provided in 4, in Section 5 pertains to the discussion while Section 6 concludes the paper.

## 2 Model formulation

The problem of optimal investment consumption investigates the most favorable approach an investor can have to utilize their funds, aiming to maximize their discounted satisfaction while minimizing potential risks or losses. This scenario arises when the investor is presented with a limited selection of investment alternatives, the money can be saved in the bank account that is the risk free asset (bond), invested in the risky asset (stock market) and used for consumptions and also for health expenses.

We consider a retiree's investment over a time interval [0,T] after they have retired. The financial market consists of only two assets: a risk free asset which is a bond with price process *B* and a risk asset which is a stock with price process *S*. The time evolution of the price of the riskless bond is described as,

(1) dB(t)=rB(t)dt,r>0.

The price of the stock follows Geometrical Brownian motion, satisfying,

(2) dS(t)=αS(t)dt+σS(t)dW(t),t>0

where α(>r) is the rate of return and *σ* is the volatility, W(t) is the Wiener process. The condition α>r guarantees considerable profit compared with the risky free asset.

The retiree consumes wealth X(t) at a non negative rate C(t) and also spends the money at a non negative rate H(t). They distribute the remaining amount of wealth between the two assets in time *t* and the remaining for health expenses. The wealth dynamics are

(3) dX(t)=ϕ(t)dS(t)+ψ(t)dB(t)

Where ϕ,ψ being number of shares of risky assets and bond respectively held by an investor. From equation (1) and (2), respectively, it follows that,

(4) $$\frac{dB(t)}{B(t)}=rdt$$

and from equation (3) it follows that,

(5) dS(t)=S(t)(αdt+σdW(t)).

When *π* is the amount of wealth in risky asset, from equation (3) it follows that,

(6) π(t)=ϕ(t)S(t),ψ(t)B(t)=X(t)−π(t)=X(t)−ϕ(t)S(t).

Specifically, the retiree in this financial market model may transfer funds from one account to another at no costs. Hence, under these suppositions using equations (3) and (6) the retiree's wealth X(t) at time *t* changes according to the following model,

(7) $$dX(t)=\phi (t)dS(t)+\psi (t)dB(t)=\frac{\pi (t)}{S(t)}dS(t)+\frac{X(t)-\pi (t)}{B(t)dB(t)}.$$

Hence, equation (7) will transform into the following model,

(8) dX(t)=(rX(t)+π(t)(α−r))dt+σdW(t).

When *π* is the proportion of risky asset, then it follows that,

(9) $$\pi (t)=\frac{\phi (t)S(t)}{X(t)},\;\phi (t)=\frac{\pi (t)X(t)}{S(t)},\;\psi (t)=\frac{X(t)(1-\pi (t)}{B(t)}.$$

Therefore, using equation (9) we transform the wealth equation as,

(10) $$d(X(t)=\phi (t)dS(t)+\psi (t)dB(t)=\frac{\pi (t)X(t)}{S(t)}dS(t)+\frac{X(t)(1-\pi (t)}{B(t)}dB(t).$$

As a result, equation (10) is simplified as,

(11) dX(t)=X(t)((r+π(t)(α−r))dt+σdW(t)).

Considering equations (8) and (11), then we have the following equation,

(12) dX(t)=[rX(t)+(α−r)π(t)−C(t)−H(t)]dt+π(t)X(t)σdW(t),0≤t≤T.

Finally, equation (12) can be simplified further and gives,

(13) dX(t)=rX(t)dt+[(α−r)π(t)]dt−C(t)dt−H(t)dt+σX(t)π(t)dW(t)

with initial condition $X(0)=x_{0},0\leq t\leq T$. The problem is bounded by the fact that the quantities π(t),H(t),C(t) and X(t) must be all positive, ∀t≥0. X(t) is positive due to the fact that the retiree can not be insolvent. Therefore equation (13) represents the wealth equation of the retiree at any time *t* based on the expenditures and investments.

### 2.1 Stochastic optimal control model

The retiree consumes C(t) on the basic and other luxury needs while H(t) is for the health expenditures. They aim to find a strategy which consists of π(t) for stock trading, a positive consumption rate C(t), a positive health expenditure H(t) in order to maximalize the expected utility. Let Γ={(π(t),C(t),H(t)):0≤t≤T} be the set of all feasible investment-consumption proportions with health expenditures. For any combination (π(t),C(t),H(t)), the equation (13) has a specific solution. Theoretically, retirees want to invest their wealth to have the greatest expected benefit, namely, maxEU(C(t)U(H(t){U(XT)}, where U(.) is a concave and smoothly differentiable utility function on (−∞,∞) and E is the expectation operator. The expectation operator is defined as,

(14) $$E[U(x)]=\sum P(x_{i})U(x_{i}),$$

where $P(x_{i})$ is the probability of outcome $x_{i}$ and $U(x_{i})$ is the associated utility. Using equation (14) the objective expected utility function is as follows,

(15) $$E\{[\mu \int_{0}^{T}e^{-\beta t}U(C(t))U(H(t))dt]+(1-\mu )e^{-\beta T}U(X_{T})\},$$

where *β* is the subjective discount. The parameter *μ* in equation (15) ascertains the relative importance of the intermediate consumption and the terminal wealth. Since U(.) is strictly concave there exists a unique optimal trading strategy (π(t),C(t),H(t)) satisfying equation (13) [17]. For a particular feedback function π(t) it can be defined as the Markov property. Our purpose is to maximize the retiree's expected utility during their lifetime, where an infinite time horizon is presumed. We get the following goal function which is referred to as the value function,

(16) $$V(x,t)=\underset{(\pi (t),C(t),H(t))\in A}{Sup}E\{[\mu \int_{0}^{T}e^{-\beta t}U(C(t))U(H(t))dt]+(1-\mu )e^{-\beta T}U(X_{T})\},\;\beta >0,X(t)=x,$$

with boundary condition given by

(17) $$V(t,x)=(1-\mu )e^{-\beta T}U(x),V(t,0)=0.$$

The value function defined by equation (16) is the upper definite bound of the expected utility, so it reaches the optimal value under the stochastic constraint [17].

We apply the method of Bellman's optimality principle in order to deduce the Hamilton- Jacobi- Bellman (HJB) equations for the value function and examine the optimal investment, health expenditure and consumption strategy respectively. If the HJB equation has a solution, then we will get the desired optimal control by using the Verification theorem as follows,

Theorem 1 *Suppose that we have two functions*H(t,x)*and*g(t,x)*, such that*

*1.* *H is sufficiently integrable, and solves the HJB equation*$\frac{\partial H}{\partial t}(t,x)+\underset{u\in U}{Sup}\left\{F(t,x,u)+A^{u}H(t,x)\right\}=0,\;\forall (t,x)\in (0,T)\times R^{n}$*.*
  $H(T,x)=\Phi (x),\;\forall x\in R^{n}$*.*
*2.* *The function g is an admissible control law.*
*3.* *For each fixed*(t,x)*, the supremum in the expression*$\underset{u\in U}{Sup}\left\{F(t,x,u)+A^{u}H(t,x)\right\}$*is attained by the choice*u=g(t,x)*.*

*Then the following hold:*

*1.* *The optimal value function V to the control problem is given by*V(t,x)=H(t,x)*.*
*2.* *There exists an optimal control law***u***, and in fact*u(t,x)=g(t,x)*.*

We consider the power utility function which is defined by,

(18) $$U(x)=\frac{x^{\eta }}{\eta },\eta <1,\eta \neq 0.$$

Using Bellman's optimality principle, we obtain the HJB equation which is satisfied by the value function V(x) for equation (13) as follows,

(19) $$\frac{\partial V}{\partial t}+\underset{(\pi (t),C(t),H(t))\in A}{Sup}\{\mu e^{-\beta t}U(C(t)U(H(t))+rx\frac{\partial V}{\partial x}+[(\alpha -r)\pi (t)]\frac{\partial V}{\partial x}-C(t)\frac{\partial V}{\partial x}-H(t)\frac{\partial V}{\partial x}+\frac{1}{2}\sigma^{2}\pi^{2}(t)\frac{\partial^{2}V}{\partial x^{2}}\}=0.$$

Simplifying equation (19) gives,

(20) $$\frac{\partial V}{\partial t}+\underset{(\pi (t),C(t),H(t))\in A}{Sup}\{\mu e^{-\beta t}U(C(t))U(H(t))+[rx-C(t)+(\alpha -r)\pi (t)-H(t)]\frac{\partial V}{\partial x}+\frac{1}{2}\sigma^{2}\pi^{2}(t)\frac{\partial^{2}V}{\partial x^{2}}\}=0,$$

where

(21) $$\frac{\partial V}{\partial t}(x,t),\frac{\partial V}{\partial x}(x,t),\frac{\partial^{2}V}{\partial x^{2}}(x,t)$$

represent first order partial derivative with respect to *t*, first order partial derivative with respect to *x*, second order partial derivative with respect to *x* respectively. Hence, equation (20) is the derived Hamilton- Jacobi- Bellman equation.

## 3 Qualitative analysis of the model

Now we need to find the explicit solution of the Hamilton-Jacobi- Bellman equation which is given in equation (20).

We need to differentiate the HJB equation with respect to π(t).

Optimal value conditions based on the first derivative are given by,

(22) $$\pi^{⁎}(t)=\frac{r-\alpha }{\sigma^{2}}\frac{\frac{\partial V}{\partial x}}{\frac{\partial^{2}V}{\partial x^{2}}}.$$

Or, written more compactly,

(23) $$\pi^{⁎}(t)=\frac{(r-\alpha )V_{x}}{\sigma^{2}V_{xx}}.$$

Therefore, we define $U^{\prime }(C^{⁎}(t))U(H(t))$ and $U^{\prime }(C(t))U(H^{⁎}(t))$ as utility function of consumption and utility function of health expenditure respectively. So using equation (20) we get the following utility functions,

(24) $$U^{\prime }(C^{⁎}(t))U(H(t))=\frac{1}{\mu }e^{\beta t}V_{x},\;\;U^{\prime }(C(t))U(H^{⁎}(t))=\frac{1}{\mu }e^{\beta t}V_{x}.$$

Consequently, introducing equations (23) and (24) into (20), we obtain the following equation,

(25) $$\frac{\partial V}{\partial t}+\mu e^{-\beta t}U(C^{⁎}(t))U(H^{⁎}(t))+[rx-C^{⁎}(t)-H^{⁎}(t)]V_{x}-\frac{{\left(\alpha -r\right)}^{2}{\left(V_{x}\right)}^{2}}{\sigma^{2}V_{xx}}+\frac{{\left(r-\alpha \right)}^{2}{\left(V_{x}\right)}^{2}}{2\sigma^{2}V_{xx}}=0.$$

We define $k=\frac{1}{2}\frac{{\left(\alpha -r)\right)}^{2}}{\sigma^{2}}$ in equation (25) and it becomes,

(26) $$\frac{\partial V}{\partial t}+\mu e^{-\beta t}U(C^{⁎}(t))U(H^{⁎}(t))+(rx-C^{⁎}(t)-H^{⁎}(t))V_{x}-K\frac{{\left(V_{x}\right)}^{2}}{V_{xx}}=0.$$

In this instance, we've observed that the stochastic control problem has undergone a transformation into a challenging non-linear second-order partial differential equation, rendering it highly intricate to solve.

Thus, we choose the power utility function for our analysis to obtain explicit solutions to equation (26).

From equation (26), we guess a solution with the following structure,

(27) $$V(t,x)=e^{-\beta t}\frac{x^{\eta }}{\eta }f(t,x),\;\;f(T,x)=1-\mu .$$

Hence, it follows that,

(28) $$\frac{\partial V}{\partial t}=e^{-\beta t}\frac{x^{\eta }}{\eta }(-\beta f+f_{t}),\;\;\frac{\partial V}{\partial x}=e^{-\beta t}(x^{\eta -1}f+\frac{1}{\eta }x^{\eta }f_{x}),\;\;\frac{\partial^{2}V}{\partial x^{2}}=e^{-\beta t}x^{\eta }[(\eta -1)\frac{f}{x^{2}}+2\frac{f_{x}}{x}+\frac{1}{\eta }f_{xx}].$$

Consequently, we need to substitute $V_{x}$ and $V_{xx}$ from equation (28) into equation (23) and yields the following equation,

(29) $$\pi^{⁎}(t)=\frac{(r-\alpha )\eta f+xf_{x}}{\eta \sigma^{2}(\eta -1)\frac{f}{x}f_{x}+f_{xx}}.$$

Now we need to solve the consumption policy $C^{⁎}(t)$ and the Health investment policy $H^{⁎}(t)$. Since $U(x)=\frac{x^{\eta }}{\eta }$ then it implies that $U(C)=\frac{C^{\eta }}{\eta }$.

We define $U^{\prime }(C^{⁎}(t))$ and $U^{\prime }(H^{⁎}(t))$ as utility functions for consumption and health expenditure respectively. From the definitions the following are the derived utility functions,

(30) $$U^{\prime }(C^{⁎}(t))=C^{\eta -1},\;\;U^{\prime }(C^{⁎}(t))=\frac{1}{\mu }e^{\beta t}V_{x},\;\;\mu^{-1}x^{\eta -1}f(t,x)+\mu^{-1}\frac{x^{\eta }}{\eta }\frac{\partial f}{\partial x}={\left(C^{⁎}(t)\right)}^{\eta -1},\;\;\mu^{-1}x^{\eta -1}[f+\frac{1}{\eta }xf_{x}]={\left(C^{⁎}(t)\right)}^{\eta -1},\mu^{\frac{1}{1-\eta }}x{\left[f+\frac{x}{\eta }f_{x}\right]}^{\frac{1}{\eta -1}}=C^{⁎}(t).$$

Similarly, we have the following,

(31) $$U(H)=\frac{H^{\eta }}{\eta },\;\;U^{\prime }(H^{⁎}(t))=H^{\eta -1},\;\;U^{\prime }(C(t))U(H^{⁎}(t))=\frac{1}{\mu }e^{\beta t}V_{x},\;\;\mu^{-1}x^{\eta -1}f(t,x)+\mu^{-1}\frac{x^{\eta }}{\eta }\frac{\partial f}{\partial x}={\left(H^{⁎}(t)\right)}^{\eta -1},\;\;\mu^{\frac{1}{1-\eta }}x{\left[f+\frac{x}{\eta }f_{x}\right]}^{-\frac{1}{\eta -1}}=(H^{⁎}(t)).$$

Consequently, from equation (31) we find $U(C^{⁎}(t))U(H(t))$ and $U(C(t))U(H^{⁎}(t))$ respectively as follows,

(32) $$U(C^{⁎}(t))U(H(t))=\frac{{\left(C^{⁎}(t)\right)}^{\eta }}{\eta },=\frac{1}{\eta }{\left({\left[\mu^{\frac{1}{1-\eta }}xf+\frac{x}{\eta }f_{x}\right]}^{\frac{-1}{1-\eta }}\right)}^{\eta },=\frac{1}{\eta }{\left[\mu^{\frac{1}{1-\eta }}xf+\frac{x}{\eta }f_{x}\right]}^{\frac{-\eta }{1-\eta }},=\frac{1}{\eta }{\left[\mu^{\frac{1}{1-\eta }}xf+\frac{x}{\eta }\right]}^{\frac{\eta }{\eta -1}},=U(C(t))U(H^{⁎}(t)).$$

Hence, substituting the partial derivatives $V_{t},V_{x},V_{xx},U(C^{⁎}(t))U(H^{⁎}(t)),C^{⁎}(t)$ and $H^{⁎}(t)$ from equations (20) and (24) into equation (26) results into,

(33) $$e^{-\beta t}\frac{x^{\eta }}{\eta }(f_{t}-\beta f)+\mu e^{-\beta t}{\left(\frac{1}{\eta }{\left[\mu^{\frac{1}{1-\eta }}xf+\frac{x}{\eta }\right]}^{\frac{\eta }{\eta -1}}\right)}^{2}+\;rxe^{-\beta t}(x^{\eta -1}f+\frac{x^{\eta }}{\eta }f_{x})-\mu^{\frac{1}{1-\eta }}x{\left[f+\frac{x}{\eta }f_{x}\right]}^{\frac{1}{\eta -1}}\;e^{-\beta t}(x^{\eta -1}f+\frac{x^{\eta }}{\eta }f_{x})-\mu^{\frac{1}{1-\eta }}x{\left[f+\frac{x}{\eta }f_{x}\right]}^{\frac{1}{\eta -1}}e^{-\beta t}(x^{\eta -1}f+\frac{x^{\eta }}{\eta }f_{x})-\frac{{\left(\alpha -r\right)}^{2}{\left[e^{-\beta t}(x^{\eta -1}f+\frac{x^{\eta }}{\eta }f_{x})\right]}^{2}}{2\sigma^{2}e^{-\beta t}x^{\eta }((\eta -1)\frac{f}{x^{2}}+2\frac{f_{x}}{x}+\frac{1}{\eta }f_{xx})}=0$$

Using the approach used by Gao [11], we apply the method of power transformation and variable substitution. So letting

(34) $$f(t,x)=g(t,y),\;y=x^{-2\sigma }$$

We get,

(35) $$\frac{\partial f}{\partial t}=\frac{\partial g}{\partial t},\;\;\frac{\partial f}{\partial x}=\frac{\partial g}{\partial y}(-2\sigma )x^{-2\sigma -1},\;\;\frac{\partial^{2}f}{\partial x^{2}}=\frac{\partial^{2}g}{\partial y^{2}}4\sigma^{2}x^{-4\sigma -2}+\frac{\partial g}{\partial y}(-2\sigma )(-2\sigma -1)x^{-2\sigma -2}.$$

Introducing these derivatives (34) and (35) into (33) we obtain

(36) $$e^{-\beta t}\frac{x^{\eta }}{\eta }(\frac{\partial g}{\partial t}-\beta g)+\mu e^{-\beta t}{\left(\frac{1}{\eta }{\left[\mu^{\frac{1}{1-\eta }}xg+\frac{x}{\eta }\right]}^{\frac{\eta }{\eta -1}}\right)}^{2}+\;rxe^{-\beta t}(x^{\eta -1}g+\frac{x^{\eta }}{\eta }\frac{\partial g}{\partial y}(-2\sigma )x^{-2\sigma -1})-\mu^{\frac{1}{1-\eta }}x{\left[g+\frac{x}{\eta }\frac{\partial g}{\partial y}(-2\sigma )x^{-2\sigma -1}\right]}^{\frac{1}{\eta -1}}e^{-\beta t}(x^{\eta -1}g+\frac{x^{\eta }}{\eta }\frac{\partial g}{\partial y}(-2\sigma )x^{-2\sigma -1})-\mu^{\frac{1}{1-\eta }}x{\left[g+\frac{x}{\eta }\frac{\partial g}{\partial y}(-2\sigma )x^{-2\sigma -1}\right]}^{\frac{1}{\eta -1}}e^{-\beta t}(x^{\eta -1}g+\frac{x^{\eta }}{\eta }\frac{\partial g}{\partial y}(-2\sigma )x^{-2\sigma -1})-\frac{{\left(\alpha -r\right)}^{2}{\left[e^{-\beta t}(x^{\eta -1}g+\frac{x^{\eta }}{\eta }\frac{\partial g}{\partial y}(-2\sigma )x^{-2\sigma -1})\right]}^{2}}{2\sigma^{2}e^{-\beta t}x^{\eta }((\eta -1)\frac{f}{x^{2}}+2\frac{\partial g}{\partial y}(-2\sigma )x^{-2\sigma -1}x+\frac{1}{\eta }\frac{\partial^{2}g}{\partial y^{2}}4\sigma^{2}x^{-4\sigma -2}+\frac{\partial g}{\partial y}(-2\sigma )(-2\sigma -1)x^{-2\sigma -2})}=0.$$

Furthermore, we use the following approach that involves alteration of variables. We presume that,

(37) $$g(t,y)={\left[h(t,y)\right]}^{1-\eta },\;h(T,y)={\left(1-\mu \right)}^{\frac{1}{1-\eta }}.$$

Then

(38) $$\frac{\partial g}{\partial t}=(1-\eta )h^{-\eta }\frac{\partial h}{\partial t},\;\;\frac{\partial g}{\partial y}=(1-\eta )h^{-\eta }\frac{\partial h}{\partial y},\;\;\frac{\partial^{2}g}{\partial y^{2}}=(1-\eta )(-\eta )h^{-\eta -1}{\left(\frac{\partial h}{\partial y}\right)}^{2}+(1-\eta )h^{-\eta }\frac{\partial^{2}h}{\partial y^{2}}.$$

Substituting equation (38) into equation (36) we have,

(39) $$e^{-\beta t}\frac{x^{\eta }}{\eta }((-\eta )h^{1-\eta }\frac{\partial h}{\partial t}-\beta h^{1-\eta })+\mu e^{-\beta t}{\left(\frac{1}{\eta }{\left[\mu^{\frac{1}{1-\eta }}xh^{1-\eta }+\frac{x}{\eta }\right]}^{\frac{\eta }{\eta -1}}\right)}^{2}+rxe^{-\beta t}(x^{\eta -1}h^{1-\eta }+\frac{x^{\eta }}{\eta }(1-\eta )h^{-\eta }\frac{\partial h}{\partial y}(-2\sigma )x^{-2\sigma -1})-\mu^{\frac{1}{1-\eta }}x{\left[h^{1-\eta }+\frac{x}{\eta }(1-\eta )h^{-\eta }\frac{\partial h}{\partial y}(-2\sigma )x^{-2\sigma -1}\right]}^{\frac{1}{\eta -1}}e^{-\beta t}(x^{\eta -1}h^{1-\eta }+\frac{x^{\eta }}{\eta }(1-\eta )h^{-\eta }\frac{\partial h}{\partial y}(-2\sigma )x^{-2\sigma -1})-\mu^{\frac{1}{1-\eta }}x{\left[h^{1-\eta }+\frac{x}{\eta }(1-\eta )h^{-\eta }\frac{\partial h}{\partial y}(-2\sigma )x^{-2\sigma -1}\right]}^{\frac{1}{\eta -1}}e^{-\beta t}(x^{\eta -1}h^{1-\eta }+\frac{x^{\eta }}{\eta }(1-\eta )h^{-\eta }\frac{\partial h}{\partial y}(-2\sigma )x^{-2\sigma -1})-\frac{{\left(\alpha -r\right)}^{2}{\left[e^{-\beta t}(x^{\eta -1}h^{1-\eta }+\frac{x^{\eta }}{\eta }(1-\eta )h^{-\eta }\frac{\partial h}{\partial y}(-2\sigma )x^{-2\sigma -1})\right]}^{2}}{2\sigma^{2}e^{-\beta t}x^{\eta }((\eta -1)\frac{f}{x^{2}}+\tau +\frac{1}{\eta }(1-\eta )(-\eta )h^{-\eta -1}{\left(\frac{\partial h}{\partial y}\right)}^{2}+(1-\eta )h^{-\eta }\frac{\partial^{2}h}{\partial y^{2}}4\sigma^{2}x^{-4\sigma -2}+\lambda )}=0,$$

with $\;h(T,y)={\left(1-\mu \right)}^{\frac{1}{1-\eta }}$, where,

(40) $$\lambda =(1-\eta )h^{-\eta }\frac{\partial h}{\partial y}(-2\sigma )(-2\sigma -1)x^{-2\sigma -2}$$

and,

(41) $$\tau =2(1-\eta )h^{-\eta }\frac{\partial h}{\partial y}(-2\sigma )x^{-2\sigma -1}x$$

Therefore, equation (39) is the partial differential equations obtained.

Observing that the equation remains a linear second-order partial differential equation, solving it directly remains a formidable challenge. Taking inspiration from Liu's method, we endeavor to match a solution to the equation (39) and we have the following Lemma:

Lemma 1 *Assume that*$h(t,y)=\mu^{\frac{1}{1-\eta }}\int_{t}^{T}\overset{˜}{h}(u,y)du+{\left(1-\mu \right)}^{\frac{1}{1-\eta }}\overset{˜}{h}(t,y)$*is a solution of*(39)*; then one can prove that*$\overset{˜}{h}(t,y)$*satisfies the equation:*

(42) $$e^{-\beta t}\frac{x^{\eta }}{\eta }((1-\eta ){\overset{˜}{h}}^{-\eta }\frac{\partial \overset{˜}{h}}{\partial t}-\beta {\overset{˜}{h}}^{1-\eta })+\mu e^{-\beta t}{\left(\frac{1}{\eta }{\left[\mu^{\frac{1}{1-\eta }}x{\overset{˜}{h}}^{1-\eta }+\frac{x}{\eta }\right]}^{\frac{\eta }{\eta -1}}\right)}^{2}+rxe^{-\beta t}(x^{\eta -1}{\overset{˜}{h}}^{1-\eta }+\frac{x^{\eta }}{\eta }(1-\eta ){\overset{˜}{h}}^{-\eta }\frac{\partial \overset{˜}{h}}{\partial y}(-2\sigma )x^{-2\sigma -1})-\mu^{\frac{1}{1-\eta }}x{\left[{\overset{˜}{h}}^{1-\eta }+\frac{x}{\eta }(1-\eta ){\overset{˜}{h}}^{-\eta }\frac{\partial \overset{˜}{h}}{\partial y}(-2\sigma )x^{-2\sigma -1}\right]}^{\frac{1}{\eta -1}}e^{-\beta t}(x^{\eta -1}{\overset{˜}{h}}^{1-\eta }+\frac{x^{\eta }}{\eta }(1-\eta ){\overset{˜}{h}}^{-\eta }\frac{\partial \overset{˜}{h}}{\partial y}(-2\sigma )x^{-2\sigma -1})-\mu^{\frac{1}{1-\eta }}x{\left[{\overset{˜}{h}}^{1-\eta }+\frac{x}{\eta }(1-\eta ){\overset{˜}{h}}^{-\eta }\frac{\partial \overset{˜}{h}}{\partial y}(-2\sigma )x^{-2\sigma -1}\right]}^{\frac{1}{\eta -1}}e^{-\beta t}(x^{\eta -1}h^{1-\eta }+\frac{x^{\eta }}{\eta }(1-\eta )h^{-\eta }\frac{\partial \overset{˜}{h}}{\partial y}(-2\sigma )x^{-2\sigma -1})-\frac{{\left(\alpha -r\right)}^{2}{\left[e^{-\beta t}(x^{\eta -1}{\overset{˜}{h}}^{1-\eta }+\frac{x^{\eta }}{\eta }(1-\eta ){\overset{˜}{h}}^{-\eta }\frac{\partial \overset{˜}{h}}{\partial y}(-2\sigma )x^{-2\sigma -1})\right]}^{2}}{2\sigma^{2}e^{-\beta t}x^{\eta }((\eta -1)\frac{f}{x^{2}}+\tau +\frac{1}{\eta }(1-\eta )(-\eta ){\overset{˜}{h}}^{-\eta -1}{\left(\frac{\partial \overset{˜}{h}}{\partial y}\right)}^{2}+(1-\eta ){\overset{˜}{h}}^{-\eta }\frac{\partial^{2}\overset{˜}{h}}{\partial y^{2}}4\sigma^{2}x^{-4\sigma -2}+\lambda )}=0,$$

*with*$\;\overset{˜}{h}(T,y)=1$*,*

*where,*

(43) $$\lambda =(1-\eta ){\overset{˜}{h}}^{-\eta }\frac{\partial \overset{˜}{h}}{\partial y}(-2\sigma )(-2\sigma -1)x^{-2\sigma -2}$$

*and,*

(44) $$\tau =2(1-\eta ){\overset{˜}{h}}^{-\eta }\frac{\partial \overset{˜}{h}}{\partial y}(-2\sigma )x^{-2\sigma -1}x$$

### 3.1 Proof

We define,

(45) $$\nabla h(t,y)=-e^{-\beta t}\frac{x^{\eta }}{\eta }\beta {\overset{˜}{h}}^{1-\eta }+\mu e^{-\beta t}{\left(\frac{1}{\eta }{\left[\mu^{\frac{1}{1-\eta }}x{\overset{˜}{h}}^{1-\eta }+\frac{x}{\eta }\right]}^{\frac{\eta }{\eta -1}}\right)}^{2}+rxe^{-\beta t}(x^{\eta -1}{\overset{˜}{h}}^{1-\eta }+\frac{x^{\eta }}{\eta }(1-\eta ){\overset{˜}{h}}^{-\eta }\frac{\partial \overset{˜}{h}}{\partial y}(-2\sigma )x^{-2\sigma -1})-\mu^{\frac{1}{1-\eta }}x{\left[{\overset{˜}{h}}^{1-\eta }+\frac{x}{\eta }(1-\eta ){\overset{˜}{h}}^{-\eta }\frac{\partial \overset{˜}{h}}{\partial y}(-2\sigma )x^{-2\sigma -1}\right]}^{\frac{1}{\eta -1}}e^{-\beta t}(x^{\eta -1}{\overset{˜}{h}}^{1-\eta }+\frac{x^{\eta }}{\eta }(1-\eta ){\overset{˜}{h}}^{-\eta }\frac{\partial \overset{˜}{h}}{\partial y}(-2\sigma )x^{-2\sigma -1})-\mu^{\frac{1}{1-\eta }}x{\left[{\overset{˜}{h}}^{1-\eta }+\frac{x}{\eta }(1-\eta ){\overset{˜}{h}}^{-\eta }\frac{\partial \overset{˜}{h}}{\partial y}(-2\sigma )x^{-2\sigma -1}\right]}^{\frac{1}{\eta -1}}e^{-\beta t}(x^{\eta -1}h^{1-\eta }+\frac{x^{\eta }}{\eta }(1-\eta )h^{-\eta }\frac{\partial \overset{˜}{h}}{\partial y}(-2\sigma )x^{-2\sigma -1})-\frac{{\left(\alpha -r\right)}^{2}{\left[e^{-\beta t}(x^{\eta -1}{\overset{˜}{h}}^{1-\eta }+\frac{x^{\eta }}{\eta }(1-\eta ){\overset{˜}{h}}^{-\eta }\frac{\partial \overset{˜}{h}}{\partial y}(-2\sigma )x^{-2\sigma -1})\right]}^{2}}{2\sigma^{2}e^{-\beta t}x^{\eta }((\eta -1)\frac{f}{x^{2}}+\tau +\frac{1}{\eta }(1-\eta )(-\eta ){\overset{˜}{h}}^{-\eta -1}{\left(\frac{\partial \overset{˜}{h}}{\partial y}\right)}^{2}+(1-\eta ){\overset{˜}{h}}^{-\eta }\frac{\partial^{2}\overset{˜}{h}}{\partial y^{2}}4\sigma^{2}x^{-4\sigma -2}+\lambda )}.$$

Then (39) can be rewritten as,

(46) $$\frac{\partial h(t,y)}{\partial t}+\nabla h(t,y)=0,\;\;h(T,y)={\left(1-\mu \right)}^{\frac{1}{\left(1-\eta \right)}}.$$

Then, it follows that,

(47) $$\frac{\partial h(t,y)}{\partial t}=-\mu^{\frac{1}{1-\eta }}\overset{˜}{h}(t,y)+{\left(1-\mu \right)}^{\frac{1}{1-\eta }}\frac{\partial \overset{˜}{h}(t,y)}{\partial t}\;\;=\mu^{\frac{1}{1-\eta }}[\int_{t}^{T}\frac{\partial \overset{˜}{h}(u,y)}{\partial u}du-\overset{˜}{h}(T,y)]+{\left(1-\mu \right)}^{\frac{1}{1-\eta }}\frac{\partial \overset{˜}{h}(t,y)}{\partial t},\;\;\nabla h(t,y)=\mu^{\frac{1}{1-\eta }}\int_{t}^{T}\nabla \overset{˜}{h}(u,y)du+{\left(1-\mu \right)}^{\frac{1}{1-\eta }}.\nabla \overset{˜}{h}(t,y).$$

Further (47) is reduced to,

(48) $$\mu^{\frac{1}{1-\eta }}[\int_{t}^{T}(\frac{\partial \overset{˜}{h}(u,y)}{\partial u}+\nabla \overset{˜}{h}(u,y))du-\overset{˜}{h}(T,y)+1]+{\left(1-\mu \right)}^{\frac{1}{1-\eta }}[\frac{\partial \overset{˜}{h}(t,y)}{\partial t}+\nabla \overset{˜}{h}(t,y)]=0.$$

Then, we obtain,

(49) $$\frac{\partial \overset{˜}{h}(t,y)}{\partial t}+\nabla \overset{˜}{h}(t,y)=0,\;\;\overset{˜}{h}(T,y)=1.$$

Therefore, (39) holds.

Taking $f(t,x)=g(t,y)={\left[h(t,y)\right]}^{1-\eta }$ and their relationships into considerations, then

Since,

(50) $$\pi^{⁎}(t)=\frac{r-\alpha }{\eta \sigma^{2}}\frac{\eta f+xf_{x}}{(\eta -1)\frac{f}{x}f_{x}+f_{xx}}.$$

Introducing equation (35) into equation (29) it yields,

(51) $$\pi^{⁎}(t)=\frac{r-\alpha \eta g+x\frac{\partial g}{\partial y}(-2\sigma )x^{-2\sigma -1}}{\eta \sigma^{2}(\eta -1)\frac{g}{x}\frac{\partial g}{\partial y}(-2\sigma )x^{-2\sigma -1}+\frac{\partial^{2}g}{\partial y^{2}}4\sigma^{2}x^{-4\sigma -2}+\frac{\partial g}{\partial y}(-2\sigma )(-2\sigma -1)x^{-2\sigma -2}}.$$

After further simplification equation (51) we get,

(52) $$\pi^{⁎}(t)=\frac{r-\alpha \eta h^{1-\eta }+x(1-\eta )h^{-\eta }\frac{\partial h}{\partial y}(-2\sigma )x^{-2\sigma -1}}{-2\sigma^{3}h^{1-2\eta }x^{-2\sigma -2}(-\eta^{3}+2\eta^{2}-\eta )\frac{\partial h}{\partial y}+(-\eta^{2}-\eta )h^{-\eta -1}{\left(\frac{\partial h}{\partial y}\right)}^{2}+(1-\eta )h^{-\eta }\frac{\partial^{2}h}{\partial y^{2}}4\sigma^{2}x^{-4\sigma -2}+\rho },$$

where,

(53) $$\rho =(1-\eta )h^{-\eta }\frac{\partial h}{\partial y}(-2\sigma )(-2\sigma -1)x^{-2\sigma -2}$$

Similarly,

(54) $$C^{⁎}(t)=\mu^{\frac{1}{1-\eta }}x{\left[f+\frac{x}{\eta }f_{x}\right]}^{\frac{1}{\eta -1}}=H^{⁎}(t)$$

Further simplification of equation (54) yields,

(55) $$C^{⁎}(t)=\mu^{\frac{1}{1-\eta }}x{\left[g+\frac{x}{\eta }\frac{\partial g}{\partial y}(-2\sigma )x^{-2\sigma -1}\right]}^{\frac{1}{\eta -1}}=H^{⁎}(t)$$

Consequently, equation (55) will result into the following,

(56) $$C^{⁎}(t)=\mu^{\frac{1}{1-\eta }}x{\left[h^{1-\eta }+\frac{x}{\eta }(1-\eta )h^{-\eta }\frac{\partial h}{\partial y}(-2\sigma )x^{-2\sigma -1}\right]}^{\frac{1}{\eta -1}}=H^{⁎}(t)$$

Therefore, equations (52) and (56) represent the optimal investment, consumption and health expenditures respectively.

Remark The derived closed-form expression in (56) constitutes the overarching structure for the most favorable investment, consumption, and health strategies within the context of the specified stochastic processes, reflecting the geometric Brownian motion dynamics outlined earlier. The resulting optimal investment strategy $\pi^{⁎}(t)$ shares a resemblance to the optimal policy found in a GBM model. According to equation (56), it shows that the amount of money to be spent in health will be the same as the amount of money to be spent in consumption since $H^{⁎}(t)=C^{⁎}(t)$. Schmidt and Walters [15] found out that if a person retires five years earlier at age 60, they expect to pay 53% more for health expenses than if they can wait until age 65. Therefore, according to our study, we can conclude that a retiree has to spend 50% of their wealth to health and the remaining for consumption which is closer to other studies. So, in our study we assumed that the retiree has 100,000,000$ which he or she can use for investment, consumption and health expenditure. The amount was assumed since the retirees have no any other means apart from the lump sum they have received which they will use throughout their stay.

## 4 Sensitivity analysis

**Figure 1:**
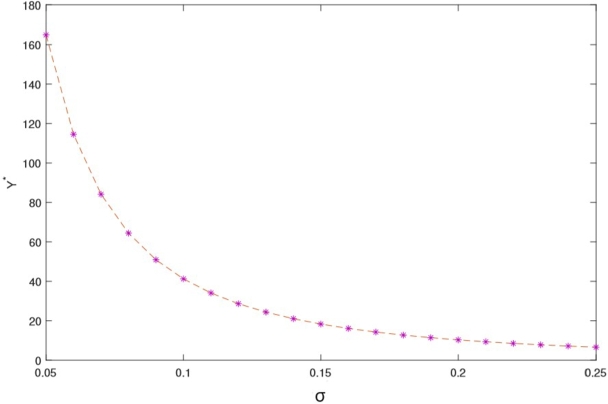
Influence of *σ* on optimal investment ratio *Y*^⁎^.

**Figure 2:**
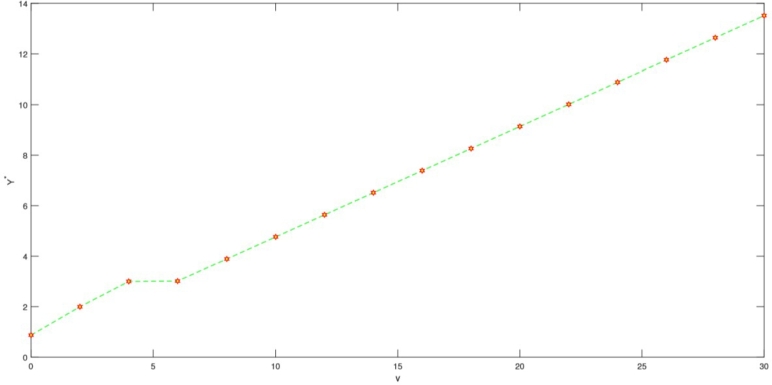
Proportional correlation between initial wealth value, health expenditure and amount invested in risky assets.

**Figure 3:**
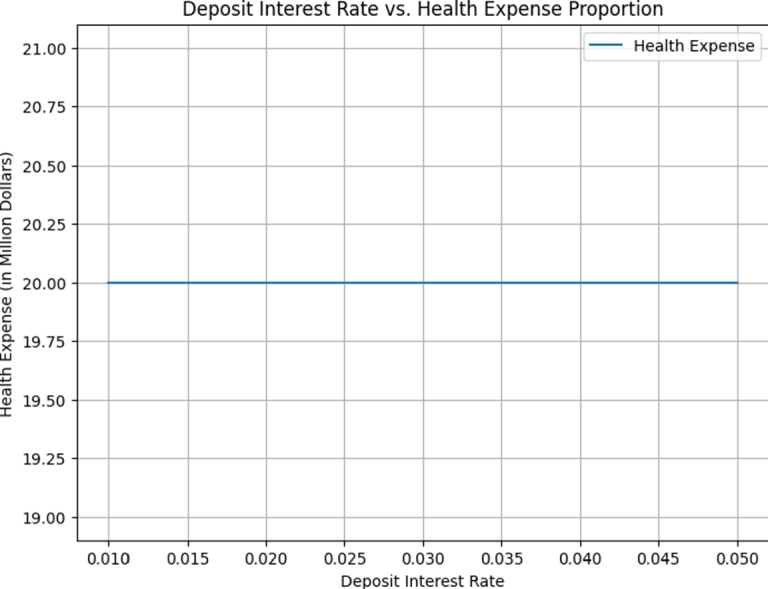
Deposit interest rate and health expense proportion.

**Figure 4:**
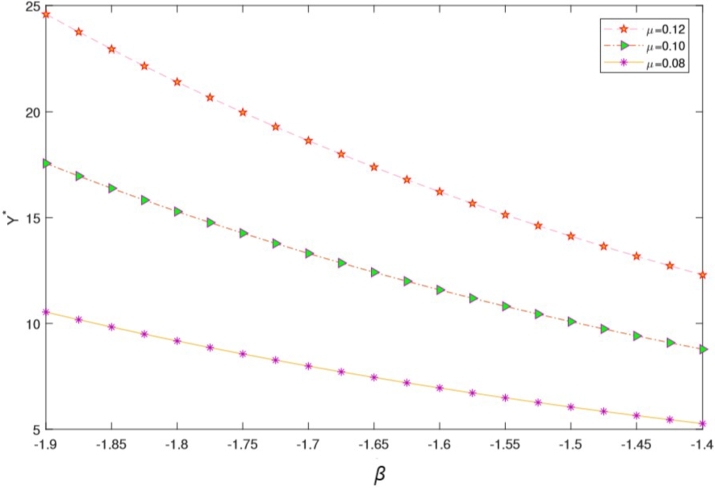
Effects of constant variance discounting coefficient and rate of return of stock on Optimal Investment strategy.

**Figure 5:**
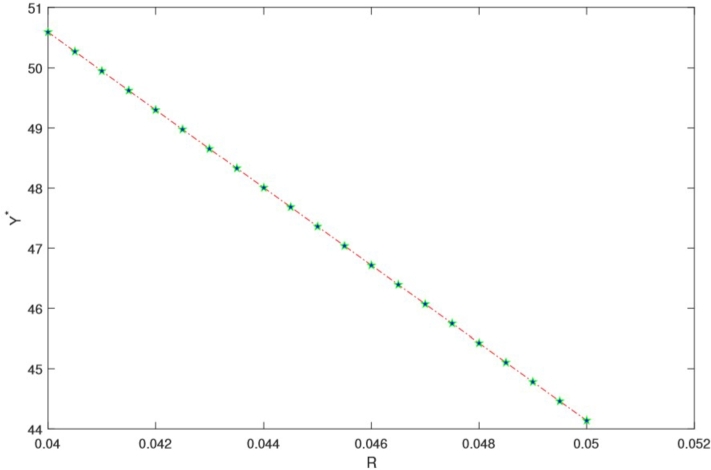
Proportion of health welfare rate *R* and wealth invested in stock.

In this section, we present a numerical example aimed at elucidating how the optimal investment, consumption, and health expenditure strategies are influenced by distinct market parameters. We focus on the context of using HARA utility with a power utility case. To ground this example, we refer to Yuen et al. [21] estimation of the Hong Kong stock option market and employ the parameter values as listed in Table 1. For the sake of generality, we assume t∈[0,T] and T=5 years. Throughout our analysis, unless explicitly indicated otherwise, the fundamental parameters are as detailed in the subsequent Table 1. We present the graphical results to show the effect of market parameters and its economic implications. The graphs are presented in Figure 1, Figure 2, Figure 3, Figure 4, Figure 5 We provide the sensitivity analysis of market parameters on the optimal investment, optimal consumption and optimal health expenditures strategy respectively. We first discuss the behavioral features related to risk aversion (*η*), volatility (*σ*) and rate of discount (*β*) characterizing contribution into investment and health. We take the initial time t=0 and assume the investor retires at T=5 years. In the financial market, the other parameters used are $r=0.03,\alpha =0.12,x_{0}=100,\beta =0.06$ and $S_{0}=67$. Further results and economic implications are summarized below:

• In Fig. 1 we compensate volatility as
  (57) $$\sigma =\alpha S_{t}^{\beta }$$
  in which there is the optimal investment strategy $Y^{⁎}$ of the retiree's account. Assuming the wealth value $dX_{t}$ at time *t* is the optimal $Y^{⁎}$, then considering the invested risky assets and the volatility in the range [0.05−0.25], it follows that equation (13) varies with it. Evidently, the increase in the parameter $\alpha S_{t}^{\beta }$ causes the reduction of $Y^{⁎}$ which signifies that investors provide fewer funds to the risky asset when the stock price fluctuations increase. From a financial point of view, this can be interpreted as the stock price fluctuation enhancing the uncertainty in the market. According to Fig. 1, as the stock price increases, there's a reduction in stock volatility, leading to diminished investment risk. Consequently, a retiree investor might feel more inclined to allocate additional funds into stocks to enhance their wealth. The distinction from Chang's approach lies in his utilization of the CEV model, incorporating an elasticity parameter [7]. This results in the observation that, under the CEV model, stock investment surpasses that of the GBM model. Both the mathematical and real-world interpretations underscore the impact of volatility on the optimal investment strategy $\left(Y^{⁎}(t)\right)$ for risky assets. These assets face the potential for devaluation. Given this circumstance, a retired individual, acting as an investor, is inclined towards more conservative investments, avoiding risky endeavors due to the potential depreciation.
• Based on Fig. 2, it's evident that a direct and positive correlation exists between the initial wealth (X(0)=v) and the investment amounts in both risky assets and health. Consequently, as the initial wealth value rises, enterprise annuity retirees tend to increase their investment in risky assets. This trend is attributed to the intrinsic connection between an enterprise employee's wealth and their risk-taking capacity. When one possesses greater wealth, they inherently possess a heightened resilience against risks.
• As evident in Fig. 3, there is a case in which the deposit interest rate is equal to the health expense proportion. We can see the case in which investing interest rate is equal to the proportionality for the health expense rate. Compared with Fig. 2 it is clear that the difference between these ratings has a significant on how the retiree invest. We can easily see that the initial wealth X(0)=v=100 is highly sustaining but retirees can consume it for instance, by taking 50% in health, invest 30% and return 20% for consumption. This is an assumption to be taken by the retiree in order not to be bankrupt. Fig. 3 is a direct result of Fig. 2. In this case *r* is the expected instantaneous rate of return of the stock. Strictly *R* is the stocking or investing rate in risky free assets over risky assets characterized by *r*. Hence, Fig. 3 has that constancy property when R=r, otherwise Fig. 3 is just Fig. 2. In Chang's scenario [7], as the risk-free interest rate *r* increases, there is a corresponding rise in the allocation to the risk-free asset, leading to a decrease in the investment allocated to stocks. Simultaneously, the cumulative anticipated wealth of the investor grows progressively larger. In contrast to this paper, it implies that the funds available for health-related expenditures will rise correspondingly, resulting in an equitable balance between health costs and deposit interest rates. However, existing literature lacks findings that demonstrate the proportional relationship between deposit interest rates and healthcare expenses. Consequently, our model had to take care of the health expense component thereby making the retirees to survive without bankruptcy until their death.
• Fig. 4 illustrates the impacts of the elasticity coefficient, *β*, and the anticipated instantaneous stock rate of return, *μ*, on the optimal investment approach. By examining Fig. 4, it becomes evident that there exists a negative correlation between the constant variance discounting coefficient and the allocation towards risky assets. This implies that when the constant variance discounting coefficient increases, seasoned enterprise annuity retirees intentionally decrease the portion of their investment allocated to risky assets. In addition, when the constant variance coefficient of the risky asset is set to a fixed value, it is found by comparison that the stock return has a certain influence on the investment ratio invested in the risky asset, and there is a positive correlation between the two. The financial background explanation is clearly apparent, the higher the expected return of the stock, the more retirees tend to invest in the risky asset.
• From Fig. 5, we see that the health expenses rate is negatively correlated with the proportion of investment in the risky asset (a retiree might choose to be inputting a proportion of investment returns in health); thus, as the health welfare rate *R* increases, the proportion of wealth invested in the stock becomes larger. These results are unique in our paper since many literatures did not compare the proportion of health welfare rate *R* and the wealth invested in stock. From a financial standpoint, this analysis suggests that an excessively high rate of investment in health could lead to significant harm to the retiree's interests. Specifically, injecting an excessive amount of capital into the risky asset is identified as a potential cause for such negative consequences. To avert more substantial financial setbacks, the retiree should steer clear of excessive risk-taking and instead adopt cautious strategies. Among these prudent approaches, a reduction in fixed expenditures can be considered (e.g., paying off a mortgage, selling a second home, throttling back support for adult children) when heading into retirement can also help reduce your reliance on investments and therefore the risk of not having enough money later.

**Table 1:** Model Parameter Values.

Symbol	Description	Value	Source
r	Interest rate of the bond	0.03%	Wang et al. [17]
*α*	Rate of return of stock	0.12%	Wang et al. [17]
*σ*	Volatility	−1	Wang et al. [17]
*S*_0_	Price process	67 (million dollars)	Wang et al. [17]
t	Initial time	0 (years)	Wang et al. [17]
T	Terminal time	5 (years)	Wang et al. [17]
*x*_0_	Initial wealth	100 (million dollars)	Assumed
*η*	Risk aversion	−2	Chang [7]
*β*	Rate of discount	0.06%	Chang [7]

## 5 Discussion

The investment returns involve a combination of a risk-free asset, two risky assets, and health expenses. The advantage lies in a comprehensive market that operates under the constraint of the inflation rate. The utility functions featuring hyperbolic absolute risk aversion (HARA) were solved analytically, and optimal strategies were developed through the application of dynamic programming and variable transformation techniques to the Hamilton-Jacobi-Bellman (HJB) equations. In contrast to Chang, their approach utilizes the CEV model—an extension of the GBM model—for formulating optimal investment and consumption strategies. The assumptions in their study align with those used in this thesis. In contrast to Tiro et al. [16] who investigated optimal investment, consumption, and portfolio selection, their study explores a scenario where a pension planner member (PPM) pursues an investment policy to fulfill specific life objectives.

The objective of the pension plan manager was to optimize the expected total wealth upon retirement, whereas our study emphasizes the health expenses incurred by a retiree without health insurance. In the study of Tiro et al. [16] the investment return process comprises risk free asset and two risky assets, and the PPM benefit lies in a complete market that is constrained by the inflation rate. A continuous time model built a portfolio among a continuum of agents (health, assets and consumption) that influence each other strategically and have mean-variance utility function. The numerical outcomes revealed diverse impacts of several economic and market parameters on the optimal strategies. The dynamics of inflation, market risk, and pricing governed the allocation between stocks, bonds, and health expenses, thus determining how to sustain the retiree's lifetime over a given period. Additionally, a graphical sensitivity analysis was employed to illustrate these trends.

From a financial perspective, our findings indicate that excessively high health investment rates could potentially jeopardize the retiree's interests by excessively channeling funds into risky assets. Furthermore, a higher expected stock return rate tends to drive retirees towards increased investment in risky assets. Our financial model aligns with contemporary investment practices, as it is practical for individuals to invest with the goal of enhancing their financial well-being during retirement.

## 6 Conclusion

Based on the results of this work, we see that the financial markets are described by the GBM. By employing stochastic optimal control methodology and applying the maximum principle to tackle the optimization challenge, we derived the trajectory of wealth and determined admissible controls. Closed-form solutions for optimal investment, consumption, and health expenditure strategies were derived in the context of power utility. We presented numerical instances to demonstrate the influence of market parameters on optimal investment, consumption, and health expenses, along with their corresponding economic implications. Consequently, we conclude how much should be put in health, investment and how much to consume so that by the specified lifetime period *T* a retiree has something at hand before registering a zero or negative value. The amount invested will help the retiree to be compensated during the retirement period.

In future research on the optimal investment and consumption problem with health expenditure, we focus on infinite series expansion method using power utility function. This is because further modifications on the conditions of utility function may lead to a simpler derivation of the HJB equation. The model can also be extended by incorporating CEV model. This model is an extension of GBM and with the same assumptions there is a need to compare results of that of GBM and CEV models. We leave these points to future research.

## CRediT authorship contribution statement

**Nelson Dzupire:** Investigation, Formal analysis, Data curation, Conceptualization. **John Mutepuwa:** Writing – review & editing, Writing – original draft, Validation, Resources, Methodology, Conceptualization.

## Declaration of Competing Interest

The authors declare that they have no known competing financial interests or personal relationships that could have appeared to influence the work reported in this paper.

## Data Availability

We declare that the data supporting the findings are available and may be obtained upon request.
